# A corpus-driven study of lexicalization models of English intransitive verbs

**DOI:** 10.3389/fpsyg.2022.1059516

**Published:** 2023-01-13

**Authors:** Xiaofang Wu, Kunxue Xiao, Min Wang, Lifen Yang

**Affiliations:** ^1^School of Foreign Studies, Chuzhou University, Chuzhou, Anhui, China; ^2^School of Foreign Studies, Guangzhou University, Guangzhou, Guangdong, China

**Keywords:** corpus-driven study, the specialized multimodal corpus of EIVs, ideal motion event category, lexicalization models of EIVs, reasons

## Abstract

This bottom-up study applied a corpus-driven approach to extract the major lexicalization models of English intransitive verbs (EIVs) through an analysis of their meanings under the Ideal Motion Event Category inspired by Talmy. A 710-photo specialized multimodal corpus of EIVs was constructed for this purpose. Data analysis showed that the major lexicalization models of EIVs include [Motion + Patient], [Motion + Manner], [Motion + Path], [Motion + Result], [Motion + Location], and [Motion + Purpose]. In-depth analysis of these models identified three major possible reasons why EIVs cannot originally take direct objects: the incorporation of [Patient], the inheritance of the intransitive feature, and the internalization of the actions expressed by EIVs. By comparing Chinese learners’ most misused EIVs with their corresponding Chinese verbs, the current study provides empirical data to illustrate why transitive misusages of EIVs might occur among Chinese learners. The findings of this study will help English learners and users better apply EIVs.

## Introduction

A sentence is usually constructed around a verb. Traditional grammar takes the “subject + verb + complement” structure as the standard clause pattern (Ungerer and Schmid, 2006, p. 176). Meanwhile, in modern linguistics and in the formal approach to language in particular, the view that the sentence is verb-centered is well-established, whereby the verb-argument construction forms the core of a sentence (Römer, 2019). Given the place held by verbs in language practice, it is assumed that studies on the various features of verbs could contribute to learners’ better understanding of verbs in a language and thus help them manipulate the language effectively.

As established by linguists, English content verbs fall into two categories: transitive and intransitive. Traditionally, verbs that take direct objects are transitive, and those that do not, are intransitive. Compared to English transitive verbs, English intransitive verbs (EIVs) are more difficult for EFL (English as a foreign language) learners to acquire because they are easily misused (Yip, 1995; Montrul, 1999; Oshita, 2000; Zhu and Wang, 2016; etc.). Oshita (2000) reports that passive unaccusative errors like “What is happened” are highly prevalent among Chinese, Japanese, and Korean learners of English. Zhu and Wang (2016) also indicate that Chinese learners of English tend to misuse EIVs as transitive verbs.

To help English learners solve this problem, an in-depth study of the various features of EIVs is required. EIV studies have mostly focused on their definition and classification. There are two major approaches to defining EIVs: the syntactic and the syntactic-semantic. Conventionally, EIVs are defined mainly from the syntactic perspective. Two well-accepted examples are: An intransitive verb is one that “intrinsically or in a particular instance, occurs without a direct object” (Trask, 1993, p. 145) and “Intransitive verbs are those verbs which do not require an object, that is, they can stand by themselves” (Al-Shujairi et al., 2015). Nevertheless, from the syntactic-semantic perspective, the syntactic approach is rather rough. As Rutherford (1998, p. 191) indicates: “Verbs exhibiting surface characteristics of intransitivity, however, are a mixed bag and can be distinguished syntactically and/or semantically.” To illustrate this point, Rutherford lists many instances like “John read/wrote/drove/etc. (*cf.* read a book/wrote a letter/drove a car).” Liu (2008) proposes the category of “pure intransitive verb,” and defines pure intransitive verbs as “They (EIVs) occur with a single argument in the subject position that typically plays the theta role of agent. They never occur with an argument in the object position.” This definition can help in the investigation of spurious EIVs like “read,” “tease,” and “anger.”

There are two classifications of EIVs: the syntactic-semantic and the semantic. Syntactic-semantically, the early representative classification comes from Perlmutter (1978). Developing the Unaccusative Hypothesis, Perlmutter classifies EIVs into unaccusatives and unergatives, and subdivides each of them according to their general semantic features. For example, unergatives have two subcategories: predicates describing willed or volitional acts and predicates describing involuntary bodily processes. The two types of EIVs were later distinguished under the framework of the argument structure with its theta theory (Burzio, 1986; Belletti, 1988; Chomsky, 1995; etc.). Nevertheless, Levin and Rappaport Hovav (1995) believe that the classification of intransitive verbs is semantically determined and syntactically displayed. Accordingly, Montrul (1999), Sorace (2000), Montrul (2005), and others, based on Perlmutter’s dichotomy, classify unaccusatives semantically into change of location, continuation of state, and existence of state, and the unergatives into uncontrolled and controlled processes. Baker (2018) proposes a hierarchy of functional heads to directly classify EIVs semantically according to the semantic classes of [±control], [±initiation], [±state], [±change], and [±telic], with semantic properties encoded in the functional heads.

The above EIV studies (i.e., those on their definition and classification) along the research path from the syntactic to the syntactic-semantic, and further from the syntactic-semantic to the semantic, feature the research tendency of semanticization. However, for a thorough understanding of EIVs, it is necessary to analyze their semantic features in depth. Although the existing semantic classifications involve their semantic properties, the terms designating each type are rather abstract (e.g., Sorace, 2000; Baker, 2018). This significantly prevents ESL (English as a second language) learners from clearly understanding why EIVs can stand alone without taking a direct object and why they could not initially be used as transitive verbs. Moreover, studies on the classification of EIVs seem to have been conducted either along an up-bottom path (e.g., Perlmutter, 1978; Sorace, 2000) because an explicit introduction to the data basis for the classification is not presented, or on the basis of small-scale data (Baker, 2018). As Baker (2018) himself states, his classification is based primarily on the analysis of a core sample of around 35 verbs.

Given the situation of the studies above, that is, the lack of semantic specificity, the seemingly up-bottom path, and the small scale of data, this study adopts a bottom-up research path to analyze the semantic structural features of EIVs based on large-scale data. To be specific, its overarching goal is to apply a corpus-driven approach (Biber, 2009) to extract the lexicalization models of EIVs from the analysis of the semantic structures of EIV data in the self-constructed specialized corpus of EIVs. Specific tasks include: re-determining the final definition of intransitive verbs (the prerequisite for the realization of the following three goals), constructing a specialized corpus of EIVs with its own prominent characteristics, extracting the lexicalization models of EIVs, and discussing the implications of the results of the extraction (i.e., why EIVs cannot initially be used as transitive verbs and why Chinese learners of English tend to use them transitively). It is hoped that the findings of this study will help English learners and users better apply EIVs.

## Research methodology

According to the inherent logic relations among the four specific tasks above, the elaboration of the research methodology in this section will move from the determination of the definition of EIVs to the methods for constructing the corpus and for generalizing the lexicalization models.

### Re-defining EIVs

As reviewed above, EIVs are mainly defined from the syntactic and syntactic-semantic perspectives. The syntactic definition of EIVs is incomplete because in certain cases, verbs— although used without objects—are still classified as transitive verbs. For example, in the conversation, “Do you understand what I say?” and “Yes, I understand,” the verb “understand” is transitive even though it is used without an object in the answer. As a distinct example, the classification of the verb “anger” in the sentences, “He angers easily” and “Mary angers him” is difficult based on the syntactic definition of intransitive verbs. Nevertheless, these problems can be solved using Liu’s (2008) syntactic-semantic definition above, according to which they are labeled “pure intransitive verbs.” Accordingly, the verbs “understand” and “anger” are not intransitive verbs because the former occurs with an argument in the object position, and the latter, with an argument playing the theta role of theme (also called patient by Liu) rather than the role of agent.

Despite this, Liu’s definition is inadequate for EIVs such as “die” and “disappear” because they never occur with a single argument in the object position, but with a single argument in the subject position that plays the theta role of experiencer. EIVs are, therefore, redefined as follows: “EIVs are the verbs that occur with a single argument only in the subject position that plays the theta role of agent or experiencer.” This definition serves as the basis for constructing the corpus of pure EIVs in the present study.

### Methods for constructing the EIV corpus

#### Sources for corpus construction

The data for the corpus were collected from two types of dictionaries: one paper dictionary, *Oxford Advanced Learner’s English-Chinese Dictionary* (*OALECD*); and three online dictionaries, *Youdao*; *Oxford Learners’ Dictionary*; and *Collins*. Their websites are:

1. http://dict.youdao.com/

2. https://www.oxfordlearnersdictionaries.com/

3. https://www.collinsdictionary.com/zh/dictionary/english

*Oxford Advanced Learner’s English-Chinese Dictionary* (*OALECD*) is created by A. S. Hornby, translated by Li Beida, and published jointly by the Commercial Press of China and Oxford University Press. The words in the dictionary are alphabetically ordered and interpreted mainly from five aspects: parts of speech, various precise English and Chinese meanings, typical examples, typical collocations, and, sometimes, necessary pictures. Its body consists of 1,774 pages. As a highly valued reference for English learners in China, it can meet their demands for communication, reading, writing and translation—it is one of the bestselling English dictionaries in China. As introduced in its publication foreword and preface, as an essential reference designed for English learners from non-English-speaking countries, its sales has been leading that of those dictionaries of the same kind. Therefore, it was selected as a key source to construct the corpus of EIVs.

Nevertheless, it should be highlighted that in this dictionary, the annotation of the transitivity of verbs is not sufficiently detailed. As the content verbs are not marked “transitive” and “intransitive,” this dictionary may cause unavoidable inconvenience in determining whether a verb should be collected or not. Therefore, three online dictionaries (*Youdao*, *Oxford Learners’ Dictionary*, and *Collins*) were selected to assist *OALECD* in determining the intransitivity of verb, as they provide explicit annotations of the intransitivity of verbs. With their assistance, the collected data were thought to be more accurate and, thus, better suited to the needs of the present study. Additionally, the meanings of verbs they provide and their illustration examples are highly detailed, which can enrich *OALECD* in these aspects. The three online dictionaries significantly improved the efficiency in collection of EIVs for study and in conducting an in-depth analysis of their semantic structures.

#### Process of constructing the EIV corpus

The corpus construction involved three steps: determining EIVs, creating files of EIVs, and building a Word file of EIVs. Prior to specifying the operation of each step, one point should be highlighted: What is examined was restricted to the first basic meaning of a verb and the relevant usages presented in the paper dictionary of *OALECD*. EIVs are used originally without direct objects; however, in extended meanings, a few of them can be used transitively. Examination of only the first basic meaning of a verb can significantly avoid the confusion of meanings and prevent the uncertainty or diversity of the lexicalization model of a given EIV.

The specific steps are as follows. The first step, critical for the corpus construction in this study, is to determine EIVs based on the newly-developed definition of pure intransitive verbs above. Specifically, the *OALECD* was examined alphabetically to target verbs and then the first meanings and relevant examples of the targeted verbs were investigated to speculate on their intransitivity. In case the intransitivity of a verb could not be determined, the three online dictionaries were consulted. If the verb was consistently labeled intransitive in two of them or all of them, it was collected. For example, the first meaning of the verb “doodle” on Page 430 of *OALECD* is “make meaningless drawings, scribbles, etc., while one is or should be thinking about something else”; it is exemplified as “Stop doodling on my notebook.” Its first basic meaning and usage suggests that it is intransitive. However, to confirm its intransitivity, the three online dictionaries were also consulted. Under the meaning that is same as, or similar to, its first basic meaning presented in *OALECD*, *Collins* demonstrates its usage by the example, “He doodled during the whole lecture”; *Youdao* presents “V-I (intransitive)” and the example “He looked across at Jackson, doodling on his notebook”; the *Oxford Learners’ Dictionary* only displays information on its usage as a noun. Considering the information provided in *Collins and Youdao* being similar to that in *OALECD*, “doodle” is deemed an intransitive verb and, thus, collected.

In developing the EIV files, a camera was used to take photos of the determined EIVs together with their meanings, examples, and attached pictures from *OALECD—*and where necessary, from the three online dictionaries—and stored as files named after the 26 letters on the computer.

Finally, the photos of the EIVs were copied into Word alphabetically. Meanwhile, the EIV words were input before their photos and numbered in Word so that the Word search function could be used for the EIVs needed in the study. The corpus of EIVs was hence constructed as a Word file.

#### Features of the EIV corpus

The features of the constructed corpus first lie in its form. Compared to traditional well-known corpora such as the *Corpus of Contemporary American English* (COCA) and the *British National Corpus* (BNC), whose data exist in text form, the corpus established for the current study contains 710 photos and is characterized by multimodal data. Specifically, the entire Word corpus is a store of EIV photos that are alphabetically ordered in Word, rendering them easily searchable. Meanwhile, the photos present various forms of information about intransitive verbs, their first basic English and Chinese meanings and related usages, their other meanings and relevant usages, and a few attached pictures. With such multimodal data, this corpus is practical for the analysis of the semantic structures and the extraction of lexicalization models of EIVs.

Secondly, because this corpus was constructed for a particular language phenomenon (EIVs), it could be regarded as a “specialized corpus” (Zibin and Altakhaineh, 2021). In total, it comprises 602 EIVs. The specific number of EIVs is shown under each letter (Table 1).

**Table 1:** Number of English intransitive verbs (EIVs).

Total number	Alphabetic order	Number of EIVs		Alphabetic order	Number of EIVs
602	A	24		N	5
	B	44		O	5
	C	51		P	39
	D	38		Q	3
	E	19		R	41
	F	32		S	117
	G	38		T	28
	H	18		U	5
	I	4		V	10
	J	12		W	27
	K	1		X	0
	L	17		Y	4
	M	19		Z	1

### Methods for extracting lexicalization models of EIVs

According to Talmy (1985, 2000, p. 24), lexicalization refers to the process in which meaning or a set of interrelated meaning components is in regular association with a morpheme. To be more explicit, it is a process of fusing or conflating the conceptual structures into a single lexical form (Brinton and Traugott, 2005, p. 19). In other words, human beings express their cognitive world by employing a morpheme in this process.

Specifically, human beings first come to know and conceptualize the world composed of things and actions through their cognitive processing, produce corresponding cognition results (i.e., concepts or categories) with certain semantic constructions in their minds, and then code or express them in language. The passage from the real world to cognition results is the process of conceptualization, whereas that from cognition results to language covers one of the processes, lexicalization. As the core part of a language, verbs are the results of human beings’ lexicalizing the motion event categories formed in the conceptualization of motion events in the real world. In other words, a verb is a lexical form that directly encodes the complex motion event structure with relatively fixed semantic constants (i.e., semantic components; Rappaport Hovav and Levin, 1998). Consequently, only after considering the motion event categories elicited by the verbs, it is possible to understand and analyze the verbs.

Regarding the basic semantic components of the motion event categories, Talmy (1985, 2000) determines six components: [Figure], [Ground], [Path], [Motion], [Manner], and [Cause]. Through the observation of the languages of the world, he extracts three major lexicalization patterns of motion verb: [Motion + Manner/Cause], [Motion + Path], and [Motion + Figure]. They form the core of Talmy’s typology theory of lexicalization patterns of motion events. Since this theory was proposed, many studies have enriched it (e.g., Slobin, 2004; Beavers et al., 2010; Croft et al., 2010). For the present study, Talmy’s basic research principles and methods for lexicalization patterns are taken as an essential theoretical reference for analyzing the lexicalization patterns of EIVs; alternatively, there are three points to be made regarding the motion event categories.

The first point pertains to the conceptualization of the semantic component of [Motion]. In Talmy’s research, this component is defined under the background of “a situation containing movement or the maintenance of a stationary location alike as a ‘motion event’” (Talmy, 1985, p. 60; Talmy, 2000, p. 25). It refers to the occurrence (moving motion) and non-occurrence (locatedness) of translational motion, excluding self-contained motion. This distinction between translational and self-contained motion implies that the concept of “motion” has its own rich connotations: either moving, continuous, and external, or stationary, instantaneous, and internal. Accordingly, in the present study, the component of [Motion] was broadly treated as equivalent to the concept of “action” that embodies the different relations among things in the world. In addition to the features above, it can be either explicit like “smile,” “stand,” and “bounce,” or implicit like “deteriorate” and “become.”

The second point relates to the number of constant semantic components covered in the motion event category. As a matter of fact, no consistency has yet been attained regarding the number of semantic components or roles of an event (Dowty, 1991; Huminski, 2021; etc.). However, according to human beings’ embodiment and cognition of the world, the occurrence of a motion often involves various relatively fixed procedures like “Who do,” “Do what,” “For whom,” “How to do,” “What tool is used to do,” “Why to do,” “Where to do,” “When to do,” “Along what path,” “For what purpose,” and “What result is produced.” Accordingly, it is thought that an ideal motion event category should conceptualize all of these procedures and contain the corresponding constant semantic components, that is, [Agent], [Experiencer], [Patient], [Beneficiary], [Manner], [Instrument], [Cause], [Location], [Time], [Path], [Purpose], and [Result].

The third point relates to the meanings of semantic components above, particularly [Experiencer] and [Path]. Despite disagreements, confusions, and boundary vagueness in the understanding of semantic components (Dowty, 1991; Huminski, 2021; etc.), each semantic component in the embodied-cognitive Ideal Motion Event Category is—to a certain extent—semantically clear because they correspond to counterpart procedures of embodied cognition. [Experiencer] and [Path] need to be explained in detail because they are understood differently from their mainstream definitions. The present study defines [Experiencer] prototypically as the entity experiencing either changes in its own inner state or condition, or else, self-contained motion, being either animate or inanimate. For example, in the sentences, “She died in 1900,” “Leather can deteriorate in damp conditions,” “The earth rotates on its axis,” and “The air moves,” the entities designated by “she,” “leather,” “the earth,” and “the air” are the experiencers. As for [Path], according to Talmy (1985, p. 61) and Talmy (2000, p. 25), it refers to the course or site that the Figure object follows or occupies. As a course has its own starting and target points, the two components of starting point (source) and target point (goal) could be conflated into the component of [Path].

**Figure 1:**
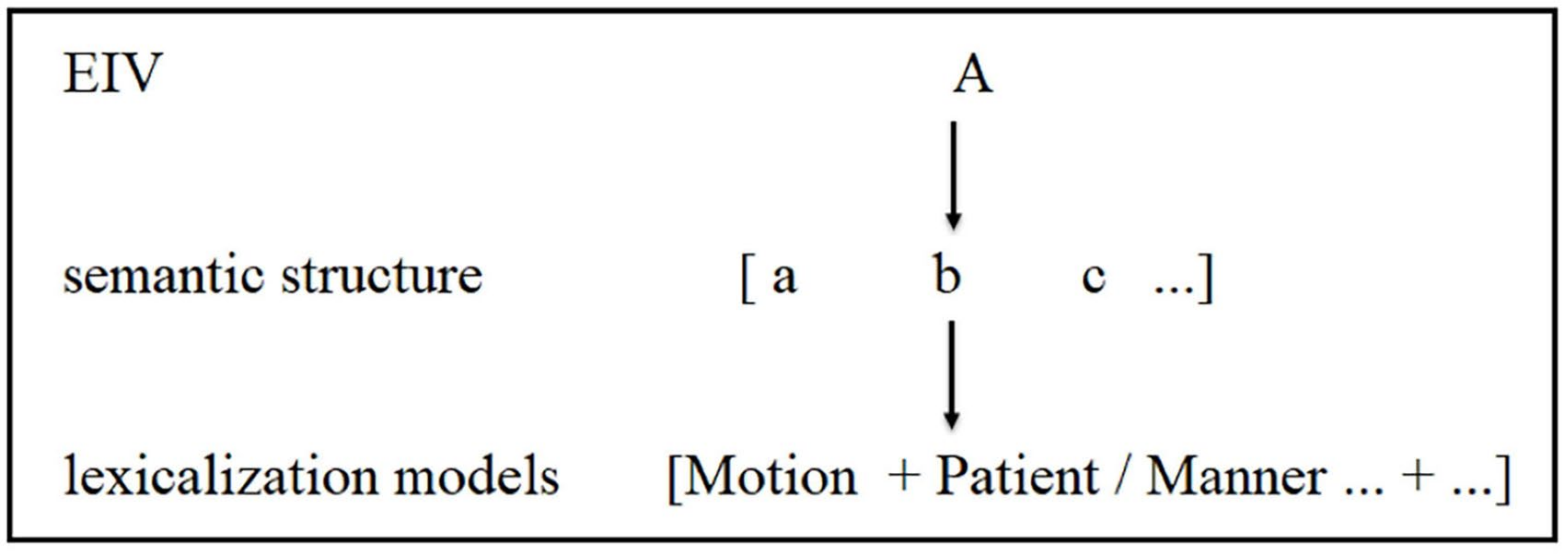
Extraction operation of the lexicalization models of EIVs.

Thus, the Ideal Motion Event Category has been created consisting of the semantic components of [Agent], [Experiencer], [Patient], [Beneficiary], [Manner], [Instrument], [Cause], [Location], [Time], [Path], [Purpose], and [Result]. With this newly-constructed Ideal Motion Event Category, the semantic structures elicited by EIVs in the specialized corpus of the present study were analyzed and the lexicalization patterns implied in these semantic structures extracted. The general extraction operation is illustrated in Figure 1.

**Figure 2:**
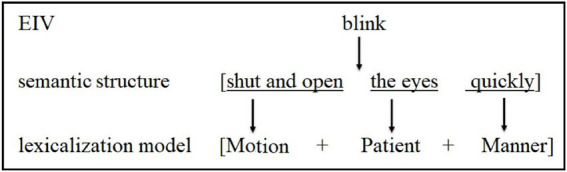
Extraction operation of the lexicalization model of “blink.”

The EIV “blink” is the example of the extraction operation. The specialized corpus of EIVs shows that its semantic structure is “shut and open the eyes quickly.” Its lexicalization model is extracted as displayed in Figure 2.

## Results: Lexicalization models of EIVs

### Presentation of lexicalization models of EIVs

By analyzing the semantic structures of collected data on EIVs, six major types of lexicalization models and several minor ones have been generalized (see Table 2).

**Table 2:** Major types of lexicalization models of EIVs.

Major types	Number	Percentage
[Motion + Patient]	284	47.18%
[Motion + Manner]	168	27.91%
[Motion + Path]	54	8.97%
[Motion + Result]	49	8.14%
[Motion + Location]	21	3.49%
[Motion + Purpose]	6	1%
Others	20	3.32%
Total: 602		

Under each major type of lexicalization model of EIVs, various subtypes are further extracted (see Table 3).

**Table 3:** Subtypes of lexicalization models of EIVs.

Major types	Subtypes	Number/percentage	Examples
Motion + Patient 284	[Motion + Patient]	179/29.73%	Bleed: lose or emit blood.
	[Motion + Patient + Manner]	32/5.32%	Proliferate: produce new growth or offspring rapidly.
	[Motion + Patient + Instrument]	24/3.99%	Profiteer: make too large a profit, esp by exploiting people in difficult times (eg, in a war or famine).
	[Motion + Patient + Manner + Cause]	15/2.49%	Gasp: take one or more quick deep breaths with open mouth because of surprise or exhaustion.
	[Motion + Patient + Purpose]	12/1.99%	Conspire: make secret plans (with others), esp to do wrong.
	[Motion + Patient + Path]	6/1%	Fart: send air from the bowels out through the anus.
	[Motion + Patient + Time]	6/1%	Doodle: make meaningless drawings, scribbles, etc, while one is or should be thinking about sth else.
	[Motion + Patient + Result]	3/0.5%	Detract: make sth seem less valuable or important.
	[Motion + Patient + Path + Manner]	3/0.5%	Snort: (usu of animals, esp horses) force air out through the nostrils with a loud noise.
	[Motion + Patient + Cause]	1/0.17%	Retire (-from sth): give up one’s regular work, esp because of age.
	[Motion + Patient + Path + Purpose]	1/0.17%	Sniff: draw air in through the nose so that there is a sound.
	[Motion + Patient + Location]	1/0.17%	Gripe: feel or cause sudden sharp pain in the stomach or intestines.
	[Motion + Patient + Path + Manner + Purpose/Time]	1/0.17%	Cough: send out air from the lungs violently and noisily, esp to clear one’s throat or when one has a cold, etc.
Motion + Manner 168	[Motion + Manner]	148/24.58%	Flounder: move or struggle helplessly or clumsily.
	[Motion + Manner + Cause]	9/1.5%	Falter: move, walk, or act hesitantly, usu because of weakness, fear, or indecision.
	[Motion + Manner + Purpose]	6/1%	Equivocate: speak in an ambiguous way to hide the truth or mislead people.
	[Motion + Manner + Location]	3/0.5%	Commute: travel regularly by bus, train, or car between one’s place of work (usu in a city) and one’s home (usu at a distance).
	[Motion + Manner + Path]	1/0.17%	Sheer: turn suddenly away from a course, topic, etc that one wishes to avoid.
	[Motion + Manner + Path + Instrument]	1/0.17%	Jump: move quickly off the ground, etc, esp up into the air, by using the force of the legs and feet.
Motion + Path 54	[Motion + Path]	36/5.98%	Emerge: come out or up (from water, etc).
	[Motion + Path + Manner]	10/1.66%	Forge: move forward steadily or gradually.
	[Motion + Path + Cause]	4/0.66%	Fall: come or go down from force of weight, loss of balance.
	[Motion + Path + Manner + Cause]	1/0.17%	Flinch: move or draw back suddenly, from shock, fear, or pain.
	[Motion + Path + Patient + Path]	1/0.17%	Defecate: push out waste from the body through the anus.
	[Motion + Path + Patient + Manner]	1/0.17%	Erupt: (of a volcano) suddenly throw out lava, etc.
	[Motion + Path + Result]	1/0.17%	Diverge: (of lines, roads, etc) separate and go in different directions, becoming further apart.
Motion + Result 49	[Motion + Result]	42/6.98%	Deteriorate: become worse in quality or condition.
	[Motion + Result + Cause]	3/0.5%	Shrink: (cause sth to) become smaller, esp because of moisture or heat or cold.
	[Motion + Result + Manner]	3/0.5%	Soak (-in sth): become thoroughly wet by being in liquid or by absorbing liquid.
	[Motion + Result + Time]	1/0.17%	Perk: become more cheerful, lively, or vigorous, esp after illness or depression
Motion + Location 21	[Motion + Location]	14/2.33%	Encamp: settle in camp.
	[Motion + Location + Instrument]	3/0.5%	Levitate: rise and float in the air, esp by means of supernatural powers.
	[Motion + Location + Time]	2/0.33%	Sojourn: stay with sb, in a place for a time.
	[Motion + Location + Cause]	2/0.33%	Drown: die in water (other liquid) because one is unable to breathe.
Motion + Purpose 6	[Motion + Purpose]	5/0.83%	Contend: struggle in order to overcome a rival, competitor, or difficulty.
	[Motion + Purpose + Time]	1/0.17%	Rally: come together, esp to make new efforts, eg, after a defeat or when there is danger, need, etc.
Others 20	[Motion]	5/0.83%	Fare: progress.
	[Motion + Time]	5/0.83%	Coincide: occur at the same time.
	[Experiencer + Motion]	5/0.83%	Blow: the air moves.
	[Motion + Cause]	4/0.66%	Writhe: twist or roll about, esp because of great pain.
	[Patient + Motion + Agent]	1/0.17%	Depend: the first thing will be affected or determined by the second.

### Interpretation of lexicalization models of EIVs

As Tables 2, 3 indicate, there are six major types of lexicalization models of EIVs: [Motion + Patient], [Motion + Manner], [Motion + Path], [Motion + Result], [Motion + Location], and [Motion + Purpose]. They, together with other minor types, will be discussed in detail in the following sections.

#### [Motion + Patient]

[Motion + Patient] is the first major lexicalization model of EIVs, numbering 284 and accounting for 47.18% of the 602 EIVs. It is defined by the two major semantic components in the semantic structures of EIVs being [Motion] and [Patient]. For example, “bleed” means “lose or emit blood.” In this meaning, the part of “lose or emit” embodies the component of [Motion], and “blood,” [Patient]. Therefore, its lexicalization model is [Motion + Patient].

In addition to the two major components conflated into the semantic structures of EIVs of this type, other minor components such as [Manner], [Instrument], [Cause], [Path], [Time], [Result], [Location], and [Purpose] are also conflated in the form of their various combinations. There are 13 such combinations: [Motion + Patient], [Motion + Patient + Manner], [Motion + Patient + Instrument], [Motion + Patient + Manner + Cause], [Motion + Patient + Purpose], [Motion + Patient + Path], [Motion + Patient + Time], [Motion + Patient + Result], [Motion + Patient + Path + Manner], [Motion + Patient + Cause], [Motion + Patient + Path + Purpose], [Motion + Patient + Location], and [Motion + Patient + Path + Manner + Purpose/Time]. Their numbers and rates are shown in Table 3.

For example, the semantic structure of “proliferate” is “produce new growth or offspring rapidly.” In the structure, “produce” embodies the semantic component of [Motion]; “new growth or offspring” specifies the component of [Patient]; “rapidly” exemplifies the component [Manner]. Therefore, the lexicalization model of “proliferate” is [Motion + Patient + Manner]. Similarly, “cough” means “send out air from the lungs violently and noisily, esp. to clear one’s throat or when one has a cold, etc.” In this structure, the parts of “send out,” “air,” “from the lungs,” “violently and noisily,” and “esp. to clear one’s throat or when one has a cold” embody, respectively, [Motion], [Patient], [Path] (fusing the starting point), [Manner], and [Purpose/Time]. Therefore, its lexicalization model is [Motion + Patient + Path + Manner + Purpose/Time].

#### [Motion + Manner]

In this model the specific parts embraced in the semantic structures of EIVs mainly embody the semantic components of [Motion] and [Manner]. For instance, in the meaning of “flounder,” “move or struggle” embodies [Motion] and “helplessly or clumsily” embodies [Manner]. As Table 3 shows, this model numbers 168; it is the second most common, immediately after the [Motion + Patient] model.

There are six subtypes: [Motion + Manner], [Motion + Manner + Cause], [Motion + Manner + Purpose], [Motion + Manner + Location], [Motion + Manner + Path], and [Motion + Manner + Path + Instrument]. This demonstrates that in addition to the two major components of [Motion] and [Manner], the semantic structures of EIVs of this type incorporate the components of [Cause], [Purpose], [Location], [Path], and [Instrument]. For example, “jump” is structured by [Motion] of “move,” [Manner] of “quickly,” [Path] of “off the ground, etc., esp. up into the air,” and [Instrument] of “by using the force of the legs and feet”; “equivocate” is semantically constructed by [Motion] of “speak,” [Manner] of “in an ambiguous way,” and [Purpose] of “to hide the truth or mislead people.”

#### [Motion + Path]

In this model, the two significant components embodied by the parts in the semantic structures of EIVs are [Motion] and [Path], with other components of [Manner], [Cause], [Patient], and [Result] occurring in certain combinations. In total, it numbers 54 and accounts for 8.97% of the 602 EIVs.

Table 2 shows its seven sub-models: [Motion + Path], [Motion + Path + Manner], [Motion + Path + Cause], [Motion + Path + Manner + Cause], [Motion + Path + Patient + Path], [Motion + Path + Patient + Manner], and [Motion + Path + Result]. For example, “emerge” is semantically constructed by [Motion] of “come” and [Path] of “out or up (from water, etc.),” whereas “flinch,” is semantically constructed by [Motion] of “move or draw,” [Path] of “back,” [Manner] of “suddenly,” and [Cause] of “from shock, fear or pain.”

#### [Motion + Result]

This model implies that the meaning of the EIV is constituted mainly by the parts embodying the components of [Motion] and [Result]. For example, in “deteriorate,” the part “become” embodies [Motion] and “worse in quality or condition” embodies [Result]. This model numbers 49 and occupies 8.14% of the 602 EIVs.

Additionally, in the semantic structures of certain EIVs of this type, other components of [Cause], [Manner], and [Time] are added to [Motion + Result]. Therefore, the other sub-models of [Motion + Result + Cause], [Motion + Result + Manner], and [Motion + Result + Time] occur. Let us consider “shrink,” in which [Cause] of “because of moisture or heat or cold” is conflated into its semantic structure in addition to [Motion] of “become” and [Result] of “smaller.” Hence, its lexicalization model is [Motion + Result + Cause].

#### [Motion + Location]

In this model, which numbers 21 and occupies 3.49% of the 602 EIVs, [Motion] and [Location] are the two major semantic components embodied in the semantic structures of EIVs. For instance, the semantic structure of “encamp” covers [Motion] of “settle” and [Location] of “in camp.”

Like the lexicalization models above, this model also embraces sub-models: [Motion + Location], [Motion + Location + Instrument], [Motion + Location + Time], and [Motion + Location + Cause]. For example, “levitate” is semantically constructed by [Motion] of “rise and float,” [Location] of “in the air,” and [Instrument] of “esp. by means of supernatural powers.”

#### [Motion + Purpose]

This model contains [Motion + Purpose] and [Motion + Purpose + Time]. It only numbers six and accounts for about 1% of the 602 EIVs. For example, the semantic structure of “contend” includes [Motion] of “struggle” and [Purpose] of “in order to overcome a rival, competitor, or difficulty,” and that of “rally” conflates [Motion] of “come together,” [Purpose] of “to make new efforts,” and [Time] of “after a defeat or when there is danger, need, etc.”

#### Others

The other types of lexicalization models of EIVs are [Motion], [Motion + Time], [Experiencer + Motion], [Motion + Cause], and [Patient + Motion + Agent]; they number, respectively, five, five, five, four, and one, and account for, respectively, 0.83%, 0.83%, 0.83%, 0.66%, and 0.17% of the 602 EIVs.

Among these, the model of [Motion] implies that the meanings of EIVs only conflate the part embodying [Motion]. For example, “fare” is semantically constituted only by [Motion] of “progress.” The [Motion + Time] model refers to the case in which the semantic constructions of EIVs conflate the parts embodying [Motion] and [Time]. For example, the meaning of “coincide” fuses [Motion] of “occur” and [Time] of “at the same time.” The [Experiencer + Motion] model may be typically exemplified by “blow.” In its semantic structure, the part of “the air” embodies [Experiencer] and “moves” embodies [Motion]. Regarding the remaining two models, typical examples are “writhe” and “depend.” In the semantic structure of “writhe,” the part of “twist or roll about” embodies [Motion] and “because of great pain” embodies [Cause]. In “depend,” “the first thing” is [Patient], with “will be affected or determined” and “by the second” being [Motion] and [Agent], respectively.

## Implications of lexicalization models of EIVs

The lexicalization models of EIVs hold many implications for the learning and use of EIVs. Here we mainly discuss two questions: (1) Why EIVs cannot initially take objects syntactically? (2) Why do Chinese learners of English tend to misuse EIVs as transitive verbs?

### Why EIVs cannot initially take objects?

Semantic features significantly influence or determine syntactic behaviors (Levin, 1993; Montrul, 1999; Saeed, 2000; Goldberg and Jackendoff, 2004; etc.). Accordingly, the lexicalization models of EIVs were analyzed and three possible reasons for why EIVs cannot originally take objects were found.

#### The incorporation of [Patient] into the semantic structure

As Tables 2, 3 show, nearly half of the EIVs are lexicalized with the [Motion + Patient] model. In other words, [Patient] is the semantic component second only to [Motion] being conflated into the semantic structures of EIVs. According to Li (2006, p. 197), [Patient] refers to “the entity undergoing the effect of some action, often undergoing some change in state.” Although [Patient] does not strictly correspond to the syntactic object, Dowty’s (1991) Argument Selection Principle states that “the argument having the greatest number of Proto-Patient entailments will be lexicalized as the direct object.” In other words, the typical patient usually occupies the syntactic position of object.

Accordingly, it might be inferred that as [Patient], which usually occurs at the syntactic position of object, has been incorporated into the semantic structures of EIVs, the given EIVs need not take objects syntactically, as doing so would lead to semantic redundancies in their syntactic structures. This is because the incorporated [Patient], functioning as invisible objects, makes the meanings of EIVs relatively independent and complete. Take “bleed” and “sniff” as examples. As Table 3 shows, their semantic structures are “lose or emit blood” and “draw air,” respectively. With the conflation of [Patient] of “blood” and “air,” their meanings have been relatively independent and complete. If “blood” and “air” are placed again after “bleed” and “sniff” in the clauses, “His head had struck the sink and was bleeding” and “She wiped her face and sniffed loudly,” the two clauses would be semantically redundant and thus, usually, unacceptable.

#### The inheritance of intransitive feature

There might be some confusion here. Those EIVs that do not incorporate [Patient] also do not take objects initially. It appears that there is no [Patient] immediately after the component of [Motion] in their lexicalization models such as [Motion + Manner], [Motion + Path], [Motion + Purpose], and [Motion + Location], but a further analysis of verbs embodying [Motion] in these models will provide some clues. Typical EIVs such as “equivocate,” “commute,” “emerge,” “contend,” and “levitate” have been selected to illustrate the issue.

**Figure 3:**
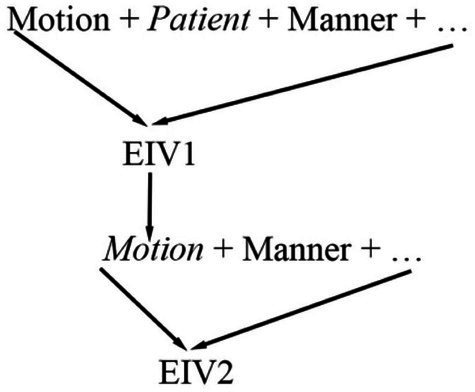
Inheritance of intransitive feature.

As Table 3 shows, the major lexicalization models of “equivocate” and “commute” are [Motion + Manner]. In their meanings, the parts representing [Motion] are two verbs: “speak” and “travel.” The specialized corpus of EIVs of the present study shows that “speak” initially means “utter words” and “travel,” “make a journey,” thereby falling under the [Motion + Patient] model. This demonstrates that they themselves are intransitive verbs. Based on this analysis, it may be inferred that in the semantic structure of a given verb, if the part embodying the constant semantic component of [Motion] is an intransitive verb, the given verb will probably also be intransitive because it inherits the intransitive feature of the intransitive verb used to explain it (see Figure 3). This given verb is EIV2 and the verb used to explain it is EIV1 in Figure 3.

This figure shows that EIV2 does not syntactically take a direct object because its meaning has indirectly incorporated the part specifying the constant semantic component of [Patient] originating from the semantic structure of EIV1. Therefore, the two EIVs, “equivocate” and “commute” are used without objects in the sentences, “Do not equivocate with me—I want a straight answer to a straight question” and “She commutes from Oxford to London every day,” just as shown in the specialized corpus of EIVs.

As for “emerge,” “contend,” and “levitate,” their lexicalization models are [Motion + Path], [Motion + Location + Instrument], and [Motion + Purpose], respectively. As Table 3 shows, in their semantic structures, [Motion] is embodied, respectively, by “come,” “struggle,” and “rise and float,” which themselves are intransitive according to the specialized corpus of EIVs. When employed to explain “emerge,” “contend,” and “levitate,” their intransitive feature might be inherited by the latter. This inheritance of intransitivity may be the reason the latter do not take objects in usages such as “The swimmer emerged from the lake,” “Several teams are contending for the prize,” and “He claims that he can levitate.”

#### The internalization of action expressed by EIVs

The analysis of the [Motion + Result] model shows that the actions expressed by EIVs of this model are internalized rather than externalized. They only involve the inner changes of states of entities and will not exert any influence on other entities, thereby syntactically taking objects out of necessity. This is consistent with Rappaport Hovav and Levin’s (1998) account of the verbs of internally caused change of state (such as “grow” and “wilt”) as different from those of externally caused change of state (such as “open” and “break”). The typical verb used to express the semantic component of [Motion] of this model is “become.” Let us consider as examples the EIVs “deteriorate” and “shrink.” Their semantic structures of “become worse in quality or condition” and “become smaller” show the inner changes of state of entities. Accordingly, they are used without objects, as in, “Leather can deteriorate in damp conditions” and “The dough shrank in the cold air.”

### Why do Chinese learners of English tend to misuse EIVs?

Zhu and Wang (2016) state that Chinese EFL learners’ transitive misuse of EIVs is mainly embodied in two forms: using them in passive voice; and putting objects immediately after them. For example, the EIVs “arrived” and “laughed” in “They just arrived a high school” and “He was laughed by others” are misused, respectively, as taking an object and in the passive voice. Various motivations for these misusages have been explored (e.g., Yip, 1995; Ju, 2000; Zhu and Wang, 2016). According to Zhu and Wang (2016), the motivation is influence from Chinese. Specifically, the Chinese verbs corresponding to the EIVs “arrive” and “laugh” are shown in (1) and (2).

**Table:** 

(1) *dao da* lit. arrive “arrive at”	(2) *xiao* lit. laugh “laugh at”

They usually take objects in Chinese, such as (3) and (4):

**Table:** 

(3) *dao da mu di di* lit. arrive the destination “arrive at the destination”	(4) *bie xiao wo le* lit. not laugh me. “Do not laugh at me.”

These transitive syntactic usages in Chinese will probably impact EFL learners’ usages of English, leading them to misuse EIVs.

The view held here is similar to that of Zhu and Wang (2016). Chinese learners’ misusages of EIVs as transitive verbs may be influenced by the usages of their corresponding Chinese verbs, constituting the result of L1 interference in learners’ second language acquisition. According to the Contrastive Analysis Hypothesis (CAH), errors are often assumed to result from learners’ transfer of their first language (Lightbown and Spada, 2006, pp. 78–79). Transfer in Odlin’s (1989, p. 27) study refers to “the influence resulting from similarities and differences between the target language and any other language that has been previously acquired.” For the identification of L1 influence, Jarvis (2000) lists three standards, whereas Ellis (2013, pp. 353–354) tabulates five types of comparison. However, Odlin (2003) indicates that “Even comparison of the use of a particular feature in the IL and L1 can provide reliable evidence of transfer effects” (cited in Ellis, 2013, p. 353). Selinker (1992) emphasizes the comparison of learners’ first language and interlanguage performances (cited in Jarvis, 2000). Specifically, if learners of the same L1 background perform congruently in their L1 and interlanguage behaviors, it could be regarded as evidence of L1 interference (Jarvis, 2000).

To verify the assumption that Chinese learners’ transitive misusages of EIVs are probably influenced by those of relevant Chinese verbs, the semantic structures and syntactic usages of Chinese verbs corresponding to the EIVs that are most frequently misused by Chinese learners have been examined. In Zhu and Wang’s (2016) study, four such EIVs were collected from *Spoken and Written English Corpus of Chinese Learners*, namely, “happen,” “arrive,” “live,” and “come.” The data in the specialized multimodal corpus of EIVs show that the lexicalization models of “happen” and “come” are [Motion] and [Motion + Path], respectively, whereas that of the others is [Motion + Patient]. As analyzed in section Why EIVs cannot initially take objects?, their intransitivity might be motivated by the incorporation of [Patient] and the inheritance of intransitive feature.

However, according to the authoritative Chinese dictionary edited by Feng (2010), *XIANDAI HANYU YONGFA CIDIAN* (*Contemporary Chinese Usage Dictionary*), the Chinese verbs corresponding to these four EIVs usually take objects syntactically. They are defined as “the predicate taking object” in this dictionary (see Table 4).

**Table 4:** Meanings and usages of Chinese verbs corresponding to “happen,” “arrive,” “live,” and “come.”

Chinese verbs	Semantic structures	Syntactic usages	Examples
*fa sheng*	*yuan lai mei you de shi chu xian le*		*fa sheng zheng chao*
lit. Happen	lit. Originally not exist things occur;		lit. *Happen* quarreling
	
“happen”	“things which originally do not exist occur”		“Quarrels *happen*.”

	*chan sheng*		
	lit. Produce		
	“produce”		
*dao da*	*dao le mou yi di dian*	*zuo dai bin yu wei yu*	*qian lai chan jia sheng hui de dai biao yi jing*
			*dao da Beijing.*
lit. Arrive	lit. Arrive a place	lit. be take object predicate	lit. Come attend meeting representatives have in succession *arrive* Beijing
“arrive at”	“arrive at a place”	“being predicate taking object”	“The representatives who will attend this meeting have *arrived at* Beijing in succession.”
*zhu*	*ju zhu, zhu shu*		*wo zhu zhe jian fang.*
lit. Live	lit. Live, lodge		lit. I *live* this room.
“live in”	“live in, lodge in”		“I live in this room.”
*lai*	*cong bie de di fang dao shuo hua ren suo zai de di fang*		*qian mian lai le yi da bang ren.*
lit. Come	lit. Form other place to speaker be located at the place		lit. The front *comes* a large group of people.
“come to”	“come from the other place to the place at which speaker is located”		“A large group of people *come* from the front.”

Table 4 shows that the semantic structures of four Chinese verbs include transitive verbs used to explain them, but do not contain fixed patients; this semantic feature determines their syntactic behavior. All of them are the predicates taking objects on the syntactic level. According to *Spoken and Written English Corpus of Chinese Learners*, the four EIVs are also used by Chinese learners as transitive verbs. Obviously, the “intra-L1-group congruity between L1 and IL performance” (Jarvis, 2000) exists in Chinese learners’ uses of EIVs. Therefore, it might be concluded that Chinese learners’ misusages of EIVs are probably motivated by Chinese L1 interference.

However, it must be admitted that Chinese L1 interference on Chinese learners’ transitive misusage of EIVs is, at best, only one possible explanation. The cognitive approach to language holds that while different people share the same faculty of cognition, they might differ tremendously in how they conceptualize the world and, thus, develop a diversity of language structures and usages. Therefore, exploration of the motivations of Chinese learners’ transitive misusages of EIVs based on the lexicalization models found in the present study should be a topic of future research.

## Conclusion and prospects for future research

Using the corpus-driven method and theoretical basis of the newly-constructed Ideal Motion Event Category inspired by Talmy’s studies, six major lexicalization models of EIVs were extracted in this study: [Motion + Patient], [Motion + Manner], [Motion + Path], [Motion + Result], [Motion + Location], and [Motion + Purpose]. Based on these six models, the probable reasons for EIVs not taking objects originally have been further explored, and the possible motivation for Chinese learners’ transitive misuse of EIVs has been analyzed. The former include the incorporation of [Patient], the inheritance of the intransitive feature, and the internalization of action expressed by EIVs; the latter is Chinese learners’ negative transfer of the transitive uses of corresponding Chinese verbs into those of relevant EIVs.

The findings of the six major models above will enrich the current research on lexicalization patterns and may also arouse further consideration of Talmy’s three-way division of lexicalization patterns. The relatively in-depth analysis of these two issues based on these models may contribute to English learners’ and users’ better understanding and application of EIVs. It might also have pedagogical implications for English teachers and help them design an effective teaching plan for EIVs.

This study has a few limitations. First, although it answers why EIVs cannot initially take objects on the ground of lexicalization models of EIVs, it does not cover the case in which EIVs are used transitively. Second, the issues of why EIVs can be used as transitive verbs and the cognitive mechanism supporting this deviant use need further exploration. Accordingly, future studies may investigate the anomalous usage of EIVs and provide a convincing explanation for this phenomenon using cognitive linguistic theories.

## Data availability statement

The raw data supporting the conclusions of this article will be made available by the authors, without undue reservation.

## Author contributions

XW provided the core idea, designed the research, and wrote the whole manuscript. KX guided the research and polished the language. MW and LY helped to collect and analyze the data. All authors contributed to the article and approved the submitted version.

## Funding

This study was sponsored by the Anhui Provincial Department of Education, “A Corpus-driven Study on Deviation Models of English Intransitive Verbs from the Perspective of Cognitive Ecolinguistics” (grant no. SK2019A0457).

## Conflict of interest

The authors declare that the research was conducted in the absence of any commercial or financial relationships that could be construed as a potential conflict of interest.

## Publisher’s note

All claims expressed in this article are solely those of the authors and do not necessarily represent those of their affiliated organizations, or those of the publisher, the editors and the reviewers. Any product that may be evaluated in this article, or claim that may be made by its manufacturer, is not guaranteed or endorsed by the publisher.
